# Engage! a pilot study of a brief behavioural activation program to promote engagement and well-being in older adults

**DOI:** 10.1371/journal.pone.0305908

**Published:** 2024-06-25

**Authors:** Sarah L. Ure, Christopher Gill, Teal Evans, Timothy D. Windsor, Julia E. T. Scott, Ruth Walker, Mary A. Luszcz, Trevor G. Mazzucchelli

**Affiliations:** 1 Curtin University, Kent St, Bentley, Western Australia, Australia; 2 Flinders University, AdelaideSouth Australia, Australia; The University of York, UNITED KINGDOM

## Abstract

Previous research has indicated the suitability of behavioural activation (BA) as an intervention for reducing depression in older adults. However, little research has investigated the potential of BA to increase active engagement and well-being in older adults. The current pilot study sought to investigate the usefulness and acceptability of BA to promote well-being in a group of non-clinical older adults. Participants (*N* = 18) aged between 65 and 86 (*M* = 77.82, *SD* = 5.59) who were retired and living independently in the community were provided a 6-week BA program predominantly delivered online. Treatment retention, self-ratings, and participants’ compliance to treatment principles indicate preliminary feasibility for the use of BA as an approach for increasing active engagement in older adult populations. Participants also provided feedback on their experiences with the program post-intervention via individual structured interviews. Thematic analysis of these data revealed that participants found the program to be beneficial in terms of increased self-awareness and social engagement, and provided several recommendations for improving acceptability of the program and workbook. The unexpected events relating to the first wave of the novel coronavirus (COVID-19) led to necessary adaptations to delivery modalities, and provided the researchers with an opportunity to investigate the use of a structured well-being program on a high-risk population during a pandemic. Our findings support the proposition that BA is a suitable intervention for increasing engagement and well-being in older adults, provide insight into adapting programs for older adults, and suggest next steps for testing intervention efficacy.

## Introduction

Globally, the general trend of increased life expectancies and lower fertility levels has led to an ageing population, with the number of people worldwide aged over 65 years expected to rise from 9% in 2019 to 16% by 2050 [1]. Population ageing is expected to result in an increased demand for health care services and facilities [2,3]. In response to these challenges, the World Health Organization [4] has released policy recommendations focussing on “active ageing”, which promotes the continuation of individuals’ participation in social, economic, and cultural affairs, according to their needs, desires, and capacity, throughout the lifespan. The WHO subsequently declared 2020–2030 to be “The decade of healthy ageing” [5] and published an action plan focussing on “bring[ing] about transformative change while building trust across generations by optimizing everyone’s opportunities for healthy ageing.” [6] In addition to its relevance to population health, there is also wide agreement within the research literature that ongoing engagement with life in older adulthood is key to the concept of “successful ageing” [7,8], and that it plays a vital role in improving quality of life and health outcomes for individuals [9]. Thus, research should focus on identifying and understanding ways in which active engagement can be achieved, in order to inform society’s preparations for the population challenges in our immediate future.

### Active engagement and older adult well-being

When older adults remain actively engaged, the impact on health and well-being outcomes is overwhelmingly positive. Adams et al. [7] conducted a meta-analysis of 42 studies published between 1995 and 2009, all of which were related to engagement and well-being in older adults. Across various domains of active engagement, they found that higher engagement was associated with better health, subjective well-being, or survival in older adults. This evidence suggests that when older adults remain actively engaged, the improved health, well-being, and survival outcomes are substantial. Conversely, lower social engagement (a key aspect of active engagement) is associated with poorer mental and physical health in older adults. There is considerable evidence for the negative impacts of social isolation on outcomes such as depression, cognitive function, cardiovascular disease, general health, and mortality [10,11]. Thus, it is important to find ways to foster and promote engagement in older adulthood as a means of supporting health and well-being.

### Engagement programs

Numerous programs have been developed for promoting active engagement in older adults, with varying levels of effectiveness [12–14]. Interventions range from focusing on productive activity, such as volunteer programs, to social and leisure activity, such as companion programs. The common theme of these programs, however, is that they are activity-specific, and may not be readily tailored to individual preferences and capacities. The literature around active engagement suggests that an activity is most likely to promote positive outcomes if the participant considers it to be personally meaningful and purposeful [15,16]. Activity-specific programs may be limited in their effectiveness because not all participants will necessarily have the capacity to do the activity being prescribed (e.g., those with functional limitations may be unable to participate in a walking group), and because prescribed activities are not sensitive to each individuals’ ideas of meaning and purpose. Indeed, when Maurici and Windsor [15] asked a group of Australian older adults directly about what activities they consider to be meaningful, participants identified a broad range of areas, including improving one’s physical health, developing relationships, serving others, and acquiring new knowledge. Considering the breadth of meaningful activity and the spectrum of capacities within older adult populations, the authors concluded that programs are more likely to promote successful ageing at a broader population level if they incorporate a person-centred approach. That is, they must consider the interests, experiences, and abilities of each individual in order to identify activities that align with their capacity and sense of meaning. One approach that is both person-centred and based on valued activities is behavioural activation (BA).

### Behavioural activation

BA was developed as a psychological therapy for the treatment of depression, and is grounded in the principles of operant conditioning and behavioural theories. These theories conceptualise the withdrawal from pleasant and valued activities observed in depression as preventing an individual from experiencing positive reinforcement, thus leading to further low mood and withdrawal [17,18]. BA is specifically designed to address this vicious cycle by introducing activity-scheduling as a means to increase engagement in valued activities, and to reconnect people with depression with the positive reinforcement of rewarding and valued activities [18,19]. Most BA methods involve a person-centred approach, whereby a facilitator works with clients to generate a list of activities that correspond to their important values, which they can then begin scheduling and completing while monitoring their mood [17,20]. BA has been shown to produce clinically significant improvements in people with depression comparable with other evidence-based approaches such as cognitive-behavioural therapy [19,21,22]. Indeed, BA is advantageous given its focus on observable behaviours rather than abstract thought processes making it more accessible for people with cognitive impairment [23].

This approach of identifying meaningful activities in order to promote positive reinforcement has led researchers to consider the potential of BA as a universal intervention for promoting well-being in both clinical and non-clinical populations. A meta-analysis conducted by Mazzucchelli et al. [24] investigated the impacts of BA on the well-being of non-clinical adults in a sample of 20 studies using BA interventions. They identified a significant effect of BA on well-being outcomes, irrespective of whether depression was indicated. These findings were supported by a randomised controlled trial conducted by Read et al. [25] investigating the efficacy of a single session of BA on improving well-being in carers. The researchers found that participants reported reduced levels of stress and an increase in behaviours consistent with their personal values, providing further evidence for the potential benefits of BA to a range of non-clinical populations.

Researchers interested in promoting health and well-being in older adults have become increasingly interested in the use of BA. Recent findings have demonstrated that BA is effective not only for treating depression in older adults, but also in improving quality of life, levels of functional ability, and mild cognitive impairment [26,27]. An evaluation of the Positive Mood and Active Life program [26], a 12-week BA program delivered to residents at risk of depression in a long-term care facility in Hong Kong, found that compared to moderate depressive symtomology at baseline, participants reported reduced depressive symptoms and improved quality of life post-intervention. While these findings support BA as an intervention for promoting health and well-being in older adults, these studies have been conducted on older adult populations that are either at-risk for depression, or have mild cognitive impairment. While research is sparse regarding the efficacy of BA in promoting active engagement in older adults from non-clinical populations, early findings are promising. Maurici and Windsor [15] reported preliminary evidence in favour of developing a BA program for widescale delivery to older adults, regardless of pre-existing depressive symptomology or level of engagement. Older adult focus groups highlighted the acceptability of a person-centred approach incorporating an individual’s values, interests, and skills in order to identify meaningful activities. It was also identified that while there were benefits to a group format, such as social interactions and co-learning, individual delivery could be more appropriate in certain instances. Consistent with these conclusions, Choi, Camaano et al. [28] investigated the use of brief individual tele-delivered BA as a means of increasing social connectedness in isolated and lonely older adults, and observed an increase in social interaction and a decrease in loneliness, depression and disability. These quantitative clinical findings were supported by qualitative findings from the same project [29], which reported participants’ unreserved approval of the tele-delivered BA program. Evidence also suggests that the benefits of brief tele-delivered BA programs for older adults are maintained to some degree over the ensuing year [30]. Thus, it appears that BA is a promising approach for use with older adults not only as a means for reducing depressive symptoms, but also for increasing engagement and well-being more broadly.

### The current study

While BA has been demonstrated as an acceptable approach for reducing depressive symptoms and increasing social connectedness in isolated older adults, there have been few, if any, studies conducted on BA programs promoting active engagement and well-being in non-clinical older adults. Thus, the purpose of the current study was to address this gap in the literature. Specifically, a pilot study was conducted in order to evaluate Engage!, a brief BA program designed to enhance active engagement and well-being among Australian older adults.

On the 11 March 2020, the World Health Organization classified COVID-19 a global pandemic [31]. The threat-containment response from the Australian Government, along with many other countries, was to restrict gatherings of more than five people, and to advise populations at greater risk of death from COVID-19, including people over 70 years of age, to self-isolate in order to protect themselves from serious illness. While these measures were enforced in the hope that Australia might avoid seeing a comparable death toll to that of China [32], the repercussions of these measures on older adult mental health are likely to have been substantial [33,34]. The data collection for the current study commenced on 27 February 2020, and was carried out during the first wave of COVID-19 in Western Australia. As such, the study’s findings relate not only to the use of BA for enhancing active engagement and well-being among older adults, but also provide lessons as to how such a program can be implemented during a pandemic.

### Aims and hypotheses

The aim of this pilot study was to assess the feasibility, acceptability, and efficacy of a brief BA intervention for older adults that enhances active engagement and well-being. We hypothesised that:

H1: It will be feasible to deliver a BA program in six sessions to enhance engagement in older adults. This will be determined according to the extent to which facilitators succeeded in delivering all program content (as assessed via session checklists).
H2: The program will be acceptable to older adults. This was determined according to the extent to which participants both attended the sessions, and endorsed items on a Client Satisfaction Questionnaire post-intervention. Acceptability was also determined by qualitative feedback obtained in a series of post-intervention focus questions.

Due to clear threats to internal validity as a result of the confounding effects of the COVID-19 pandemic on our quantitative data, our hypotheses related to potential efficacy could not be reliability tested (details of quantitative data collected may be accessed via the online supplementary materials).

Increases were to be determined by the proportion of participants who achieved reliable and clinical change on these variables assessed by self-reported measures.

## Method

The present study was approved by the Curtin University Human Research Ethics Committee (approval number: HRE2019-0694) and was performed in accordance with the ethical standards laid down in the 1964 Declaration of Helsinki and its later amendments. All participants gave their informed consent prior to their inclusion in the study. The protocol was registered with the Australian New Zealand Clinical Trials Registry (registration number ACTRN12620000126910).

### Design

The current study adopted a mixed methods approach incorporating a single group pre-test post-test design approach and the collection of qualitative data via individual interviews post-intervention. This mixed-methods approach is appropriate for pilot studies investigating the feasibility of a novel intervention.

### Recruitment and eligibility criteria

Participants were recruited via the use of a presentation and flyers at a retirement village, with people having expressed interest in being contacted in order to screen for eligibility. Although it was intended to recruit a broader, more representative, community sample, physical distancing/self-isolation measures introduced with the onset of COVID-19 disrupted further planned recruitment efforts. Individuals were eligible to participate if they were aged 65 years or over, fluent in English, fully retired, and living in the community. A prior diagnosis of dementia was the only exclusion criterion. All participants who expressed interest in the program met the inclusion criteria and went on to participate in the program.

### Participants

Participants were 18 older adults aged between 65 and 86 (*M* = 77.82, *SD* = 5.59), who reported low levels of anxiety (*M* = 5.00, *SD* = 3.60, possible range: 0–21), depression (*M* = 3.67, *SD* = 2.33, possible range: 0–21), and relatively high engagement in meaningful activity (*M* = 25.28, *SD* = 3.30, possible range: 6–30,), based on scores on the Hospital Anxiety and Depression Scale (HADS) and the Life Engagement Test (LET) respectively. Seventeen of the participants were residing in a large retirement village located in metropolitan Perth, and one was residing in the nearby community. A priori power analysis indicated 28 participants were required to capture a “medium” (*f* = 0.25) main effect for time at an alpha level of 0.05 [35]. However, our initial plan to recruit additional participants from alternative avenues was interrupted due to COVID-19 restrictions.

Thirteen women and five men participated in the program, with three male/female married couples participating together. Sixteen participants (88.9%) attended all six sessions of the program, while two participants (11.1%), both from group 1, withdrew from the program after attending session 3 (this time point coincided with the introduction of COVID-19 restrictions). Fourteen participants completed outcome measures at one week (Time 2), 15 completed outcome measures at one-month (Time 3), 12 participated in post-intervention interviews, and 13 completed outcome measures at 3-months (Time 4) after the final session.

With respect to the qualitative analysis, information power, a concept that uses study aims, sample specificity, salience of established theory, dialogue quality, and strength of analysis to inform required sample size, suggests that interviews with12 participants would generate sufficient data [36]. This was supported by research done by Guest et al. [37], who posited that approximately 90% of the total useful information can be gathered through interviews from 12 participants.

### Materials

#### Measures

We included a range of measures assessing aspects of psychosocial functioning. However, the onset of the pandemic and introduction of lockdown after pre-test meant that the validity of pre- and post-test comparisons was significantly undermined by restrictions to participants’ out-of-home activity and social contact, and our small sample. We consequently present quantitative findings just for the subset of key measures outlined below in supplementary materials and focus on participants’ qualitative responses in evaluating preliminary feasibility.

#### Life Engagement Test (LET) [38]

The LET is a six-item measure which reports a global score for meaningful activity levels using a 5-point Likert scale (1 = strongly disagree; 5 = strongly agree). The LET has been shown to have high internal consistency (average α = 0.80) and convergent and discriminant predictive validity [38].

#### Hospital Anxiety and Depression Scale (HADS) [39]

The HADS is a 14-item measure which produces two subscales for Anxiety and Depression. Participants report the severity of their symptoms (e.g., “I feel tense or wound up”) over the past week using a 4-point Likert scale. This scale has good internal consistency (Anxiety subscale *α* = 0.84, Depression subscale *α* = 0.75) when used among older adult samples [40], and convergent validity with other measures of anxiety and depression [41].

#### Demographic Questionnaire

A brief demographic survey was used to collect information about participants’ age in years, gender, marital status, languages spoken, ethnicity, education level, living arrangements, home ownership, and covering of expenses.

#### Client Satisfaction Questionnaire (CSQ)

A 19-item CSQ, adapted from Mazzucchelli, Rees et al. [42] was administered one week after the final session. The items asked participants to report their satisfaction with the program, whether or not they used the various components of the intervention, and their perception of the usefulness of the content covered in the intervention. These items were rated on a 7-point Likert scale (1 = “no, definitely not”; 7 = “yes, definitely”), with a final open-ended question included to provide an opportunity for narrative feedback.

#### Post Intervention Interview Questions

Interview questions for the verbal feedback sessions were developed using the questioning format outlined by Morgan, Krueger, and King [43]. These questions asked for both positive (e.g., “What, if any, benefits did you receive from the program?”, “What aspects of the program did you find the most useful?”) and negative (e.g., “What were the things that you did not like from the program?”, “Are there ways the program could be improved?”) feedback regarding the program’s acceptability, perceived benefits and, outcomes, as well as recommendations for future adaptations.

#### Intervention

The Engage! program was adapted from the Revised Behavioral Activation Treatment for Depression (BATD-R) [44] manual, with the primary aims being to alter the focus of the program to be around improving well-being in an older adult population, rather than treating depression. A number of key program adaptations were made (see Table 1), incorporating recommendations based on the optimisation of BATD-R [45], adapting BATD for different age groups [46], and adapting BA for older adults specifically [47]. The finalised version was provided to participants as a hard copy workbook with handouts for each week of the program.

**Table 1:** Summary of the key program adaptations and associated rationales.

Adaptation	Rationale
Information and focus on well-being rather than depression • “What is Well-Being” section in session 1	Preliminary evidence indicates BA may be an appropriate intervention for improving general well-being in older adults [15,24,25]
Consideration of values and activities relevant to older adults • Age-appropriate examples • Addition of a list of pleasant activity ideas, organised according to different life areas • Simplified life areas and values structure (reduced from five life areas of relationships, education/career, recreation/interests, mind/body/spirit, and daily responsibilities, to the three areas of me, the things that matter, and the people that matter)	Pass et al. [46] recommend consideration of values relevant to the population in question. We adapted examples within the text to reflect values commonly relevant to older adults, and included a supplementary list of pleasant activities ideas appropriate for older adults.
Consideration of physical and cognitive constraints in older adult population • Larger font size and increased space for writing in homework materials • Simplified language • Simple graphics to illustrate key points • Simplified daily monitoring forms (i.e., 5 periods in the day rather than hours) • Providing examples of completed forms	Adaptations to improving the program’s accessibility followed recommendations made by Pasterfield et al. [47] for adapting BA to older adults.
Emphasis on the function of behaviour and identifying alternative, functionally equivalent activities • Discussions of alternative activities included in relation to activity selection in the workbook	Identifying functionally equivalent activities was emphasised in the current study, given the increased likelihood of older adults ceasing particular activities due to age-related factors such as physical and cognitive decline, and stressful events.
Functional assessment of avoidance and homework non-completion • Tailored suggestions to address barriers included weekly in the workbook	Unlike Martell et al.’s [17] approach, BATD-R does not emphasise functional assessment of avoidance and homework non-completion. We incorporated a brief checklist prompting participants to consider why homework was not completed and guiding them to tailored solutions to address possible factors.

We intended to deliver the Engage! program as four 90-minute in-person group sessions, and two 30-minute telephone or video call sessions, run over a six-week period. The BATD-R protocol [44] is comprised of ten sessions, with five unique content sessions and up to five concept review sessions. Due to resource constraints, and given the evidence to suggest that treatment effects for BA can be obtained with brief interventions [25,48,49], the Engage! program was shortened to six sessions (see Table 2 for an overview of session content).

**Table 2:** Overview of session content and planned delivery.

Week	Content
**1. Well-being**	• Working as a group • What is well-being? • The activation approach to well-being • Stressful events and loss • Learning your patterns of behaviour: • Introduction to daily monitoring
**2. Life areas, values, and activities**	• Review of monitoring • Troubleshooting problems with monitoring • Life areas, values, and activities
**3. Activity selection and ranking**	• Review of monitoring • Review of life areas, values, and activities inventory • Activity selection and ranking • Daily monitoring and activity planning
**4. Reviewing achievements and seeking support**	• Review of monitoring / activities • Troubleshooting problems with activities • Seeking support from others • Daily monitoring with activity planning
**5. Reviewing achievements and modifying activities**	• Review of monitoring / activities • Troubleshooting problems with activities • Seeking support from others • Review of life areas, values, and activities • Review of activity selection and ranking • Daily monitoring with activity planning
**6. Beyond the program**	• Review of monitoring / activities • Troubleshooting problems with activities • Daily monitoring with activity planning • Review of progress • Preparing for the future

### Facilitators

The program was facilitated by a female postgraduate clinical psychologist trainee (SLU) and a male clinical psychologist registrar (CG), both of whom have previous training and experience delivering BA and groups, and undertaking qualitative research. SLU was undertaking the research to fulfil the research requirements of her Master of Psychology (Clinical) degree. CG was participating in the research as a casually employed research assistant. The facilitators participated in weekly supervision meetings facilitated by TM, a registered clinical psychologist.

### Protocol adherence

Detailed written protocols that specified the content of each session, in-session exercises to complete, and homework tasks were developed. Facilitators completed protocol adherence checklists at the end of each session to monitor content adherence. The facilitators indicated that 96.5% (SD = 15.1%) of the session content was delivered. A research assistant with an honours degree in psychology observed (via video or audio-recordings) 20% of sessions and reported 87.8% agreement with facilitator ratings (κ = 0.46).

### Procedure

Participants who met eligibility criteria were sent an information sheet and consent form, which they were required to read and complete prior to commencing. Participants then completed the pre-intervention survey measures (Time 1), either online using survey software (*n* = 10) or using a printed hard copy version (*n* = 8) depending on their preference. Hard copy versions were mailed to participants’ home addresses, with a pre-paid envelope included for them to return the measures once completed.

**Fig 1:**
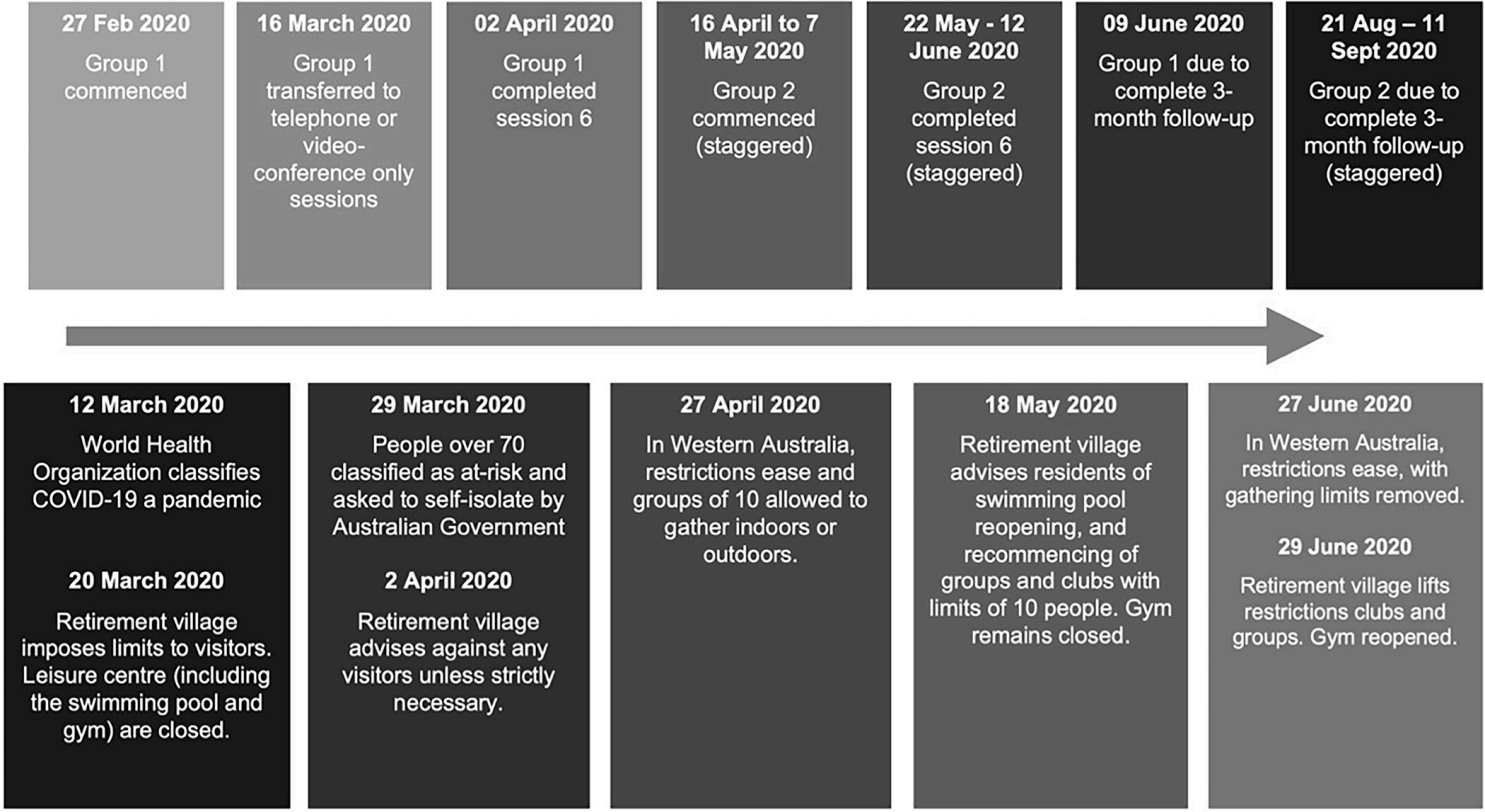
Timeline for interaction between Engage! program and outbreak of COVID-19 pandemic in Western Australia.

**Fig 2:**
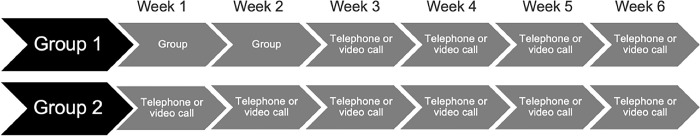
Overview of session formats used for Group 1 and Group 2.

We planned the Engage! program to be delivered using a combination of in-person group sessions and one-to-one between-session telephone calls. The first 10 participants received the initial three sessions of the program as per the planned format, with two sessions as a group (held in a function room within a retirement village), and a telephone or video call for the third. However, the introduction of government physical distancing measures to control the COVID-19 pandemic resulted in the program’s delivery being significantly impacted (see Fig 1). As such, the first 10 participants (Group 1) received subsequent sessions, and the following eight participants (Group 2) received all program sessions, via videoconferencing or, if they preferred, telephone (see Fig 2). These sessions were a mixture of small group and one-to-one sessions.

Participants took part in a single individual feedback interview using a semi-structured interview schedule with either SLU or CG one week after the final session, as well as completing outcome measures at Time 2, Time 3, and Time 4. Participants understood that the purpose of these interviews and assessments was for the research team to better understand the acceptability and impact of the program and how it could be improved for future participants. Interviews were held for 16 to 48 minutes with the average interview taking 35 minutes. Participants were assigned a unique identification code to preserve their anonymity. All electronic and hard copy data were deidentified and stored securely within Curtin University.

### Qualitative analysis

Due to threats to the internal validity of our quantitative data (as described above in the Hypotheses section), the focus of our results is on the qualitative data. Details of quantitative data collected may be accessed via the online supplementary materials.

After audio recordings of the individual feedback interviews were transcribed, qualitative data were analysed inductively by the first author (SLU) using coding reliability thematic analysis as outlined by Braun and Clarke [50]. The data analysis followed Braun and Clarke’s [51] six phases of thematic analysis. Interview transcripts were imported into qualitative analysis software, QSR NVivo 11. After reading through the transcripts in order to become familiar with the data, initial codes were generated and text extracts were grouped together according to shared codes across the whole data set. Codes were subsequently collated into possible themes and these themes were reviewed in order to determine whether they were cohesive according to the coded extracts and whole data set. A second analyst (CG) checked every code allocation for each extract, which resulted in excellent (κ = .84) inter-rater reliability. Themes were then defined and named.

Reflexivity was maintained by the research team through the data collection, analysis, and writing in a range of ways including recording, discussing, and challenging assumptions (e.g., that older adults would have a preference for spending time with other older adults, may not be interested in engaging in certain pursuits, or may not participate in video conferencing). In addition, both SLU and CG kept reflexive journals.

## Results

Due to threats to the internal validity of our quantitative data (as described above in the Hypotheses section), the focus of our results is on the qualitative data. Details of quantitative data collected may be accessed via the online supplementary materials.

### Descriptive results

Information from the demographic survey is presented in Table 3.

**Table 3:** Characteristics of study participants (N = 18).

	*M*	*SD*	*n*	%
Age (years)	77.82	5.59		
Gender (female/male)			13/5	72.22
Marital Status Married/De Facto Widowed Divorced/Separated			11 3 4	61.11 16.67 22.22
Ethnicity Australian European (including British and Irish) Southern and Eastern African			10 6 2	55.56 33.33 11.11
Education Year 8 or below Year 9, 10, 11 or equivalent Year 12 or equivalent			2 7 9	11.11 38.89 50
Living Arrangements Live alone Live with others			6 12	33.33 66.67
Expenses Can purchase most things we want Can purchase only some things we want Cannot purchase much of anything we want			13 4 1	72.22 22.22 5.56

### Program acceptability

A summary of participants’ compliance with the program, based on attendance records and responses of the 15 participants who completed the CSQ, is provided in Table 4.

**Table 4:** Percentage of participants completing each program element.

Program Element	Week of Program					
	1	2	3	4	5	6
Attended sessions	100	88.89	100	100	100	100
Read workbook	100	100	100	100	100	100
Completed workbook exercises	85.71	92.86	92.86	92.86	92.31	92.31
Planned and completed activities	84.62	92.31	84.62	100	92.86	92.86

*Note*. Session attendance based on attendance records. Other responses based on participant report as to whether they completed/used program element.

The majority (86.7%) of the participants reported being “satisfied” or “very satisfied” with the program overall, with the same percentage rating the quality of the service to be “excellent” or “good”. All of the participants reported being “satisfied” or “very satisfied” with the amount of help they received, and the majority (73.3%) reported that the program had met “almost all” or “most” of their needs. The majority (53.3%) reported that they would recommend the program to others, with the remainder reporting as either neutral (40%) or against (6.67%) recommending the program. None of the participants reported feeling that their needs were too complex to be adequately met by the program.

### Qualitative findings

Twelve participants who completed the full program participated in a feedback interview. Our thematic analysis resulted in five distinct themes, and twelve subthemes, related to the usefulness of the program (*Broad Program Benefits)*, acceptability of the program and workbook *(Barriers to Implementation* and *Workbook Structure)*, preferences for delivery method *(Program Delivery)*, and the current cohort’s characteristics (*Characteristics of the Cohort)*.

#### Broad program benefits

**Planning,** *Monitoring*, *and Doing Activities*. Participants commented on the usefulness of broader program concepts, such as the planning, monitoring, and doing activities. A 79-year-old woman commented “…when you write something down, you actually remember it better. It reinforces what you’re going to do”. The majority of participants were already highly engaged and saturated in terms of time commitments; thus, it appears that they found the program helpful for prioritising rather than increasing activities. A 77-year-old woman stated “I suppose the main thing is that [the program] makes me focus on structuring my life a bit more. I do a whole lot you see, but it helps to get the balance right”. Another participant who had retired several months before participating said:

“If not for the program, I may have doubted whether I should be doing these relaxing things as opposed to getting very busy like my husband did…. It helped me shut out the influences from the outside environment, so I am able to say ‘I’m going my way at my own pace’” (65-year-old woman)

*Self-Aware Engagement*. Another key benefit of the program was the experience of increased “Self-Aware Engagement”, referring to participants’ experiences of becoming more aware of not just the number of activities being completed, but of the quality of these activities, and whether they aligned with participants’ values. Many participants reflected on their engagement and considered the meaning of activities in relation to their values:

“It’s made me look deeper into myself rather than just watching television or just doing the same old…. It’s given me a chance to look within and say, ‘Am I really living a fulfilled life? Am I doing what I really want to do, as opposed to what I think I should do?’” (71-year-old woman)

*Social Engagement*. Social engagement was extracted from the data as an additional benefit of the program. Many participants described the activities that they were planning and doing as being socially-oriented, with an 80-year-old woman saying “I made a list of family and friends I would like to contact and those I need to contact and determined I would make contact with two each day, either a visit or phone call.” A 71-year-old woman described the program’s coverage of social support as helpful, saying “I’m able to ask people now to do things… it’s quite a nice thing to do, to ask other people to help”.

#### Barriers to implementation

*COVID-19 Disruption*. The primary barrier to implementing strategies was identified as COVID-19 (referenced 31 times in all 16 interviews). Participants described the difficulties they had with implementing the program’s tasks while their preferred activities were so restricted. “There was nothing that anyone could do unless you do it by yourself, so that really put the lid on anything else” (82-year-old woman). Participants conveyed a general sense of regret that the program had occurred alongside COVID-19 restrictions.

*“Other Barriers”*. In a number of cases participants stated that they had not completed all of the tasks, or had only done so for a certain period of the program before ceasing. A 70-year-old woman who reported a diagnosis of mild cognitive impairment stated, “No, there’s no point in scheduling anything because what I did find is that most of the time you don’t know what day it is”, indicating that further support may have been required for her to implement the strategies effectively.

#### Workbook structure

*Ease of Implementation*. Several participants commented on the ease with which they found they were able to implement the strategies in the program, with comments such as: “Every aspect of it has been quite enjoyable doing it, and it’s quite easy to do” (79-year-old woman) and “The program kept things simple and basic, lots of self-internal activities, which was right for me” (65-year-old woman).

*Formatting*. Participants favoured aspects of the workbook formatting such as the size of the text (“it’s well printed. It’s a nice size for the ages of people, nice lettering”; 86-year-old man), the way it was set out (“I think that this is quite a good way of doing them… you’ve got the daily monitoring, and then you’ve got new information… reviewing the monitoring makes you think about what you’ve written”; 79-year-old woman), and the expression of information (“What you wrote in the manual, for example “think about this” or “do this” was all well expressed and set-out nicely”; 65-year-old woman).

*Workbook Improvement*. Repetition and the length were identified as predominant areas for improving the workbook. Participants noted the repetition of both the worksheets and the daily monitoring forms. “There was a lot of repetition after several weeks of forms… so you just don’t pay the same level of attention to it” (75-year-old man). For some participants, the length of the physical workbook was a deterrent. An 86-year-old man, who initially withdrew from the program upon receiving the workbook but later re-joined, said “it can be frightening when you see the size of the book… I think the book could be kept to the minimum.”

Difficulty understanding was identified as an area for improvement, as participants displayed difficulties with comprehending some of the program’s concepts. “Someone has to be pretty willing… to comprehend what’s being asked. I just thought it’s maybe a little bit academic” (82-year-old woman). A 79-year-old woman interpreted the program as requiring she give up other enjoyable activities in order to participate fully, such as when she was offered a trip away. “To me, that was much better than sitting in here. Rather than being attached to the program for the week, I just went down and had a great time.” This comment demonstrates a significant gap in the participant’s understanding of the program’s core tenets.

#### Program delivery

*Group Format*. A predominant preference from the participants regarding delivery of the program was for a group format (referenced 39 times by 15 participants). Reasons were primarily related to the benefits of idea sharing in group contexts, with an 80-year-old woman saying “when you’re in a group, other people can give you a different answer to the way that you’ve interpreted the question.” Two participants also noted that groups may not be suitable for certain personality types, with a 79-year-old woman saying: “Some people hate groups… They might dry up in a group and be afraid to put their opinions forth.”

*Individual Telephone Calls*. Participants generally commented on having enjoyed the telephone calls, noting that the benefits of one-on-one sessions allowed for more depth within the conversations. “… the [conversation] might not have been so deep if you’ve got a group of people” (82-year-old woman). A 79-year-old woman expressed her dislike of the telephone format due to her having hearing difficulties, explaining “it’s really sometimes quite hard to hear. So, that’s the bit that I find quite difficult to do.”

*Video Calls*. We explicitly asked participants about their feelings towards video format, should it have been possible to facilitate. There was an even split between participants expressing a preference for video, and those advising that they would not be comfortable with this method. Participants favouring video commented on the increased intimacy of this medium (“I suppose it might be a little bit more intimate, if you can see somebody, you can see their reaction to questions or answers”; 79-year-old woman), while reasons for participant discomfort towards video technology ranged from a lack of confidence with technology, eye strain from using a screen, and a discomfort with being on camera.

#### Specifics of the current cohort

*Existing Engagement*. Several participants reflected on the usefulness of the program for audiences in different circumstances to themselves. Overall, the participants referred to the fact that they already led busy and active lives prior to participating, and noted that this may have impacted on how much they benefited from the program given they were already doing much of what it promoted. “I think I may be slightly outside of the people you’re aiming at because I’m fairly active….” (86-year-old man)

*Living in a Retirement Village*. Many of the participants acknowledged the fact that residing in a retirement village may inherently remove some barriers to remaining engaged in older adulthood, due to the proximity of facilities and other people with whom you might interact. One participant said “the fact that you’re in a village you can overcome a lot of that [avoidance] because everything’s right on your doorstep. So, it’s a false picture we can give from here.” (86-year-old man).

## Discussion

Our aim was to test the feasibility, acceptability, and efficacy of a brief BA intervention for older adults to enhance active engagement and well-being. While quantitative evidence supporting the efficacy of the program could not be meaningfully assessed due to the threat that COVID-19 posed to the internal validity of the study’s single group pre-test post-test design, qualitative data were gathered relating to the feasibility and acceptability of the program. This informed a number of recommended revisions to the program, as well as a discussion of limitations and future directions for the research.

The first hypothesis (H1) of the current study was that it would be feasible to deliver a brief BA program to enhance engagement and well-being in older adults in six sessions that comprised of a combination of group sessions and one-to-one phone calls. Our hypothesis was supported, as according to session checklists the facilitators delivered 96.5% of the program content to the participants.

The second hypothesis (H2) was that the program would be acceptable to older adults. This was judged by both participant responses on a client satisfaction questionnaire, and qualitative feedback obtained in an interview. Based on the questionnaire, this hypothesis was also supported, with participants demonstrating their overall acceptance of the program through their high levels of compliance with program tasks, as well as through their positive ratings of satisfaction with the program. Further support for H2 was evident from participants’ qualitative feedback captured in the theme relating to *Broad Program Benefits*, which related to the usefulness of the planning, monitoring, and doing of activities (all core features of BA), as well as the increase in both self-awareness and social engagement. Although it is only possible to speculate, the benefits for social engagement mentioned by several participants suggest that the program may have played a role in offsetting threats to social connections arising from social distancing and the pandemic for some participants [52]. Social networks have long been recognised as a key resource for health and well-being [53,54], and contemporary models of social relationships over the lifespan have highlighted the value of maintaining diverse social connections into later life [55]. These preliminary findings suggest that BA focused approaches could have a role to play in not only reducing social isolation [28], but also building on, or increasing contact frequency with existing networks.

### Recommended revisions

Participants also provided feedback on areas in which the program could be improved, which we have combined with facilitator observations in order to inform our recommended revisions for future iterations of the program (see Table 5).

**Table 5:** Summary of the recommended revisions and associated rationales.

Revision	Rationale
Increased emphasis on change Additional encouragement within workbook to either choose new activities or increase the frequency of current ones	The facilitators observed that participants would often consider the activities they already do when creating their lists of activities to schedule, and demonstrate reluctance to select activities that were new or different.
Reduction in repetition and increased structure added to workbook Removal of repeated worksheets Daily monitoring as optional from session 3 onwards. Addition of tabs in workbook for ease of navigation Addition of page-header indicating the week of the program	Repetition and workbook length was commented on by the majority of the participants as a negative aspect. The facilitators observed that participants had some difficulty with navigating the workbook due to the large number of pages, and the fact that workbook pages did not note which week of the program they related to.
Simplification of language and concepts Simplified structure (e.g., removal of life areas, leaving values identification as a stand-alone task aided by prompts such as “How do you want to be and behave in relation to your mental and physical health, and enjoying life?”) Reduction in academic language (e.g., renaming the “Life Areas, Values, and Activities Inventory” to the “My Values Worksheet”, and the renaming the “Activity Selection and Ranking” exercise to “My Activity Shortlist”)	Participants displayed some difficulties understanding the values clarification exercise in session 2 and some words used in the workbook.

### Limitations and directions for future research

The scope of the current study was limited to older adults who were generally already highly engaged and did not collect any information on experiences of more isolated and disengaged individuals. This was a limitation in terms of determining how useful and acceptable the program might be for older adults who may benefit most from programs such as this, including those aged 75 and older, among whom it is estimated that almost 20% are lonely [56]. Although our acceptability hypothesis was supported, with the majority of participants reporting satisfaction with the program, 40% of the participants were ambivalent as to whether they would recommend the program to others. While it is unclear why this discrepancy was observed, one possible explanation is that while the participants were satisfied with the program, they did not feel that they needed it. While the participants found that it increased their self-awareness, planning and self-management skills, sense of self-efficacy in terms of living a valued existence, and was, overall, “helpful”, they may not have felt that it added to or changed their lives significantly. As suggested by high scores on the LET (supplementary materials) a ceiling effect may have been operating where it was not possible to affect meaningful increases in engagement. Further, given that the participants were predominantly living in the retirement village, and their social circles were largely located within the village, it may be that the participants were unlikely to recommend the program to others who lived in the village and who had similarly high pre-existing engagement levels.

Social isolation and loneliness have been identified as an increasing risk to older adult health outcomes [10,11], and thus it is of particular importance that programs promote engagement in those older adults whose engagement, and consequently health, declines with age. This has been brought into sharp focus during the pandemic, when social distancing requirements have had substantial psychological and social impacts for many older adults [57]. Recent research has investigated the use of BA to increase social connectedness in isolated and lonely older adults [29], however data is sparse regarding the acceptability and usefulness of this approach in such populations. While the current study was able to gather information that might inform future research on engagement programs for older adults, and programs designed for older adults more broadly, it is limited in its ability to convey the perspectives of more socially isolated older adults. Future research should aim to access these less optimally engaged populations in order to determine whether the program is feasible and acceptable to those older adults who might benefit most from it.

The events of the COVID-19 pandemic significantly interrupted the execution of the current study in terms of the planned number and community representativeness of participants, delivery method, and our ability to collect quantitative data for the program’s potential to benefit older adults. We were unable to recruit from a wider range of locations, and the final number of participants was lower than intended. Of most significance was the confounding effect of COVID-19 restrictions on the levels of engagement the participants were capable of during their participation in the program. Due to the marked reduction in possibilities for activities, and increased isolation experienced by the participants due to the rules for visitation imposed by the retirement village, engagement and well-being were likely to have been significantly negatively affected during the period in which the program ran. As such, we could not collect meaningful evidence as to the potential benefits of the program on these outcomes. The program may have supported participants to maintain their activity levels, and in this way protected them from some of the deleterious effects associated with the physical distancing restrictions that were imposed during the pandemic. However, without a control group we were unable to test this.

As already noted, another consequence of the COVID-19 pandemic was that the program was not able to delivered in the hybrid group format as originally intended. It is commonly assumed that it is easier to support change in people in groups than at the individual level, in part due to the social support that groups provide [58]. Group programs have been found to reduce social isolation and loneliness in older adults [59,60]. Although videoconferencing technology permits groups to be run online, in the present study, many participants’ unfamiliarity or discomfort with this technology meant that it could not be adopted for all participants. It is an open question as to whether the predominant individual delivery of the program affected the effectiveness of the program. However, a serendipitous outcome of the present work was that it demonstrated that one-to-one telephone and videoconferencing delivery of the program appears acceptable and viable. The accessibility and efficiency of this method of delivery make it a mode of delivery that is worth pursuing in future research. This is true for psychosocial interventions such as BA generally, but especially to provide supports in the event of similar disruptive events in the future, including future epidemic or pandemics.

Digital communication technologies can play an important role in supporting healthy aging, both via stabilising contact with existing social networks, but also by facilitating connections with new supports—such as that offered by the present program. An important learning from the COVID-19 experience, including that of the present research, is the need to address the digital divide. Although it can be anticipated that successive generations will, proportionally, be increasingly digitally literate, people of older generations have had less opportunities to interact with computers, the internet, smartphones, or tablets and, as a consequence, are more likely to lack confidence using digital services such as videoconferencing software [61]. Sensitively developed community based digital training services which take into account sensory, cognitive, and physical limitations of aging are needed to help eliminate technology fears, increase older adult’s access to information and communication resources available through technology, and promote social connection and resilience [62,63].

One of the advantages of contemporary, values-based, BA is its inherent flexibility. Importantly, possible modifications do not just extend to the pacing or order of content, the manner in which the rationale is explained, or the assessment/monitoring is conducted, but also to activation assignments [62]. Participants are not limited to engaging in activities that they have enjoyed in the past (but may now be out of their reach or no longer enjoyable due to physical limitations or restrictions due to COVID). Cassar et al. [45] use the concept of “angles” to explain that there can be multiple ways of approaching a values-based goal. A participant may value being physically fit, but may no longer be able to access the gym or swimming pool. It is important to recognise that there are numerous activities consistent with this value, such as engaging in calisthenics at home, adding walking breaks to the day, cooking and eating healthy meals, experimenting with non-alcoholic cocktails, establishing good sleep habits. The concept of angles provides a framework for navigating the sometimes very real obstacles to engaging in particular activities. The challenge becomes one of generating creative values-consistent activities. Facilitators should be encouraged to stockpile examples of values and the wide range of values-consistent activities that can be shared to engage participants in ideographically-focused brainstorming discussions.

## Conclusion

To conclude, the current study found that a brief BA program to promote engagement and well-being in older adults was both feasible and acceptable. Our results indicate that overall, participant feedback supports BA’s usefulness with older adults for the purpose of increasing active engagement. Given that the number of older adults in the population is set to rise substantially over the coming decades, our study represents an important step towards adapting BA for this population, and determining its potential to improve older adult health and well-being.

## Supporting information

S1 Checklist (PDF)

S2 Checklist (DOCX)

S1 File Quantitative data collection and results. (DOCX)

S2 File Quantitative data collection and results. (XLSX)
