# Supplementary material for: Engage! a pilot study of a brief behavioural activation program to promote engagement and well-being in older adults
Source: PLoS One. 2024 Jun 25;19(6):e0305908. doi: 10.1371/journal.pone.0305908 (PMC11198748; doi:10.1371/journal.pone.0305908)
Supplement: S1 File — (DOCX) [file pone.0305908.s003.docx]

## **Supplementary material: quantitative data collection and results**

## **Method**

### Measures.

***Life Engagement Test (LET)*** (1). The outcome of meaningful activity levels was measured using the LET, a self-report measure consisting of six items (e.g., “I value my activities a lot”) measured on a 5-point Likert scale (1 = strongly disagree; 5 = strongly agree). Scoring is conducted in two steps, the first being that items 1, 3, and 5 are reverse coded, and the second being that all six items are then summed to report a global score where higher scores indicate higher levels of life engagement. The LET has been shown to have high internal consistency (average α = 0.80) and convergent and discriminant predictive validity (1).

***Scale of Positive and Negative Experience (SPANE)*** (2). The SPANE is a 12-item measure of well-being which produces a Positive Feelings subscale score (six items; e.g., “pleasant”, “contented”), a Negative Feelings subscale score (six items; e.g., “sad”, “angry”), and a balance score that can be obtained by combining the prior two together. Participants rate how much they experienced each feeling over the past 4 weeks using a 5-point Likert scale (1 = very rarely or never; 5 = very often or always). The SPANE has shown good internal consistency Valued Living α = 0.76; Life Fulfilment α = 0.89 (Negative Feelings α = 0.81; Positive Feelings α = 0.87; Balance α = 0.89; (2)) and moderate convergent validity (3)

***Satisfaction with Life Scale (SWLS)*** (6). The SWLS is comprised of five items (e.g., “I am satisfied with my life”) scored using a 7-point Likert scale (1 = strongly disagree; 7 = strongly agree), and produces a global life satisfaction score. Higher scores indicate greater satisfaction with life (4). The scale has been demonstrated to have good internal consistency (*α* = 0.84; (5), as well as convergent and discriminant validity (6).

***Hospital Anxiety and Depression Scale (HADS)*** (7). The HADS is a 14-item measure which produces an Anxiety subscale and a Depression subscale. Participants report the severity of their symptoms over the past week using a 4-point Likert scale (e.g., “I feel tense or wound up”). This scale has good internal consistency (Anxiety subscale *α* = 0.84, Depression subscale *α* = 0.75) when used among older adult samples (8) and convergent validity with other measures of anxiety and depression (9).

***Goal Adjustment Scale (GAS)*** (10). The GAS is a measure of coping flexibility, and is comprised of 10-items scored using a 5-point Likert-type scales (1 = strongly disagree; 5 = strongly agree). Participants report their typical reactions towards having to stop their pursuit of important life goals, with the mean score of four items producing a goal disengagement subscale (e.g., “It’s easy for me to reduce my effort towards the goal”), and the mean score of six items producing a goal reengagement subscale (e.g., “I start working on other new goals”). Higher scores indicate that participants find it easier to disengage from unfeasible goals and reengage in new goals. The goal disengagement and goal reengagement subscales have both been demonstrated to have good internal consistency (α = 0.84 and α = 0.86 respectively (10)) and good convergent validity (11).

***Behavioral Activation for Depression Scale-Short Form (BADS-SF)*** (12). The BADS-SF is a 9-item self-report scale measuring activation and avoidance. Participants rate how much they experienced each feeling over the past week using a 7-point Likert scale (0 = not at all; 6 = completely). Scoring is conducted in two steps, the first being that items one, six, seven and eight are reverse coded, and the second being that all nine items are then summed to report a global score, where higher scores indicate higher levels of activation. Six items can be summed together to produce an activation subscale, and three items to produce an avoidance subscale. The scale has demonstrated good internal consistency for the total scale (α = 0.82) and two subscales (Activation α = 0.81; Avoidance α = 0.82), as well as construct validity (12).

***Engaged Living Scale-Short Form (ELS-SF)*** (13). The ELS-SF is a 9-item measure that is used to assess participation in valued activities. Participants use a 5-point Likert scale (1 = completely disagree; 5 = completely agree) to indicate the extent to which they agree with a series of statements (e.g., “I make choices based on my values, even if it is stressful.”). The scale is comprised of two subscales, Valued Living and Life Fulfilment. This scale has been shown to have good internal consistency for the total scale (α = 0.88) and two subscales (Valued Living α = 0.76; Life Fulfilment α = 0.89; Trindade et al., 2016), and has demonstrated both convergent and discriminant validity (13).

Quantitative Analytic Approach

Participants’ scores on the LET, SPANE, SWLS, GAS, BADS-SF, and ELS-SF were assessed using the reliable change index. This can be calculated by determining the degree to which a participant changes on an outcome variable divided by the standard error of difference between the pre-test and post-test scores.

$$RCI=\frac{x_{post}-x_{pre}}{\sqrt{2S_{E}^{2}}}$$

Cronbach’s α of each measure was used as the reliability parameter using Ley’s formula (14) for calculating the standard error of difference between the two measurements.

$$S_{E}=SD\sqrt{1-r_{xx}}$$

Reliable change that is significant at the *p* > .05 level can be interpreted as having occurred where the reliable change index is greater than an absolute value of 1.96 (15, 16), indicating that a real change has been observed rather than any fluctuations within the measurement instrument.

# **Results**

## **Reliable and Clinically Significant Change**

Table S1 displays the number and percentage of participants who showed a positive or negative reliable change. The percentage of participants scoring in the clinical range at pre- and postintervention on the LET, SPANE, SWLS, HADS, GAS, BADS-SF, and ELS-SF is also reported in Table S1 and illustrates movement out of the clinical range. For the LET, SPANE, SWLS, HADS, GAS, BADS-SF, and ELS-SF, clinical cut-off scores were 1±*SD* the mean of the normative group (16).

The results are difficult to interpret given the intersection between the time period in which the data was collected and the impact of COVID-19 on the constructs represented by this data.

**Table S1**

*Reliable Change Indices and Clinical Change (*N *= 18)*

| Measure | Reliably Improved  % (*n*) | | | Reliably Worse  % (*n*) | | |  | Clinical Range  % (*n*) | | | |
| --- | --- | --- | --- | --- | --- | --- | --- | --- | --- | --- | --- |
|  | Time 2 | Time 3 | Time 4 | Time 2 | Time 3 | Time 4 |  | Time 1 | Time 2 | Time 3 | Time 4 |
| LET | 5.6 (1) | 5.6 (1) | 5.6 (1) | 0 (0) | 0 (0) | 5.6 (1) |  | 0 (0) | 11.1 (2) | 5.6 (1) | 5.6 (1) |
| SPANE |  |  |  |  |  |  |  |  |  |  |  |
| Positive Feeling | 11.1 (2) | 16.7 (3) | 0 (0) | 16.7 (3) | 5.6 (1) | 5.6 (1) |  | 5.6 (1) | 0.0 (0) | 0.0 (0) | 0.0 (0) |
| Negative Feeling | 5.6 (1) | 11.1 (2) | 5.6 (1) | 0.0 (0) | 5.6 (1) | 0.0 (0) |  | 5.6 (1) | 5.6 (1) | 5.6 (1) | 5.6 (1) |
| SWLS | 11.1 (2) | 11.1 (2) | 11.1 (2) | 16.7 (3) | 5.6 (1) | 11.1 (2) |  | 11.1 (2) | 0.0 (0) | 5.6 (1) | 0.0 (0) |
| HADS |  |  |  |  |  |  |  |  |  |  |  |
| Anxiety | 5.6 (1) | 5.6 (1) | 11.1 (2) | 0.0 (0) | 0.0 (0) | 0.0 (0) |  | 16.7 (3) | 11.1 (2) | 5.6 (1) | 16.7 (3) |
| Depression | 0.0 (0) | 0.0 (0) | 0.0 (0) | 5.6 (1) | 0.0 (0) | 0.0 (0) |  | 5.6 (1) | 11.1 (2) | 0.0 (0) | 5.6 (1) |
| GAS |  |  |  |  |  |  |  |  |  |  |  |
| Goal Disengagement | 5.6 (1) | 22.2 (4) | 22.2 (4) | 33.3 (6) | 22.2 (4) | 11.1 (2) |  | 27.8 (5) | 11.1 (2) | 38.9 (7) | 22.2 (4) |
| Goal Reengagement | 16.7 (3) | 22.2 (4) | 16.7 (3) | 0.0 (0) | 0.0 (0) | 5.6 (1) |  | 11.1 (2) | 5.6 (1) | 11.1 (2) | 5.6 (1) |
| BADS-SF |  |  |  |  |  |  |  |  |  |  |  |
| Activation | 16.7 (3) | 16.7 (3) | 16.7 (3) | 5.6 (1) | 0.0 (0) | 11.1 (2) |  | 0.0 (0) | 0.0 (0) | 0.0 (0) | 0.0 (0) |
| Avoidance | 0.0 (0) | 0.0 (0) | 0.0 (0) | 5.6 (1) | 5.6 (1) | 5.6 (1) |  | 5.6 (1) | 5.6 (1) | 5.6 (1) | 5.6 (1) |
| ELS-SF |  |  |  |  |  |  |  |  |  |  |  |
| Valued Living | 0.0 (0) | 0.0 (0) | 0.0 (0) | 0.0 (0) | 5.6 (1) | 0.0 (0) |  | 0.0 (0) | 0.0 (0) | 0.0 (0) | 0.0 (0) |
| Life Fulfilment | 16.7 (3) | 5.6 (1) | 0.0 (0) | 5.6 (1) | 5.6 (1) | 0.0 (0) |  | 44.4 (8/) | 38.9 (7) | 11.1 (2) | 33.33 (6) |

*Note.* LET = Life Engagement Test, SPANE = Scale of Positive and Negative Experience, SWLS = Satisfaction with Life Scale, HADS = Hospital Anxiety and Depression Scale, GAS = Goal Adjustment Scale, BADS-SF = Behavioural Activation for Depression Scale-Short Form, ELS-SF = Engaged Living Scale-Short Form; Time 1 = before the first session, Time 2 = after the final session, Time 3 = one-month after the final session, Time 4 = 3-months after the final session.

# **References**

1. Scheier MF, Wrosch C, Baum A, Cohen S, Martire LM, Matthews KA, et al. The Life Engagement Test: assessing purpose in life. J Behav Med. 2006;29(3):291-8.

2. Diener E, Wirtz D, Tov W, Kim-Prieto C, Choi D-w, Oishi S, et al. New well-being measures: short scales to assess flourishing and positive and negative feelings. Social Indicators Research. 2010;97(2):143-56.

3. Jovanovic V. Beyond the PANAS: Incremental validity of the Scale of Positive and Negative Experience (SPANE) in relation to well-being. Personality and Individual Differences. 2015;86:487-91.

4. Diener E, Emmons RA, Larsem RJ, Griffin S. The Satisfaction With Life Scale. J Pers Assess. 1985;49(1):71-5.

5. Steger MF, Frazier P, Oishi S, Kaler M. The Meaning in Life Questionnaire: assessing the presence of and search for meaning in life. Journal of Counseling Psychology. 2006;53(1):80-93.

6. Pavot W, Diener E. Review of the Satisfaction With Life Scale. Dordrecht: Dordrecht: Springer Netherlands; 2009. p. 101-17.

7. Zigmond AS, Snaith RP. The Hospital Anxiety and Depression Scale. 1983;67(6):361-70.

8. Roberts MH, Fletcher RB, Merrick PL. The validity and clinical utility of the Hospital Anxiety and Depression Scale (HADS) with older adult New Zealanders. Int Psychogeriatr. 2014;26(2):325-33.

9. Bjelland I, Dahl AA, Haug TT, Neckelmann D. The validity of the Hospital Anxiety and Depression Scale: An updated literature review. J Psychosom Res. 2002;52(2):69-77.

10. Wrosch C, Scheier MF, Miller GE, Schulz R, Carver CS. Adaptive self-regulation of unattainable goals: goal disengagement, goal reengagement, and subjective well-being. Pers Soc Psychol Bull. 2003;29(12):1494-508.

11. Wrosch C, Amir E, Miller GE. Goal adjustment capacities, coping, and subjective well-being: the sample case of caregiving for a family member with mental illness. J Pers Soc Psychol. 2011;100(5):934-46.

12. Manos RC, Kanter JW, Luo W. The Behavioral Activation for Depression Scale–Short Form: development and validation. Behav Ther. 2011;42(4):726-39.

13. Trindade IA, Ferreira C, Pinto-Gouveia J, Nooren L. Clarity of personal values and committed action: development of a shorter engaged living scale. Journal of Psychopathology and Behavioral Assessment. 2016;38(2):258-65.

14. Ley P. Quantitative aspects of psychological assessment: an introduction. Gerald Duckworth; 1972.

15. Jacobson NS, Truax P. Clinical significance: a statistical approach to defining meaningful change in psychotherapy research. Jouranl of Consulting and Clinical Psychology. 1992;59(1):12-9

16. Wise EA. Methods for analyzing psychotherapy outcomes: a review of clinical significance, reliable change, and recommendations for future directions. J Pers Assess. 2004;82(1):50-9.
